# Relevance of structural defects to the mechanism of mechanical deformation in metallic glasses

**DOI:** 10.1038/s41598-023-42685-y

**Published:** 2023-09-25

**Authors:** Charles K. C. Lieou, Takeshi Egami

**Affiliations:** 1 Department of Nuclear Engineering, University of Tennessee, Knoxville, TN USA; 2 Department of Materials Science and Engineering, University of Tennessee, Knoxville, TN USA; 3 Department of Physics and Astronomy, University of Tennessee, Knoxville, TN USA; 4 Materials Science and Technology Division, Oak Ridge National Laboratory, Oak Ridge, TN USA

**Keywords:** Materials science, Physics

## Abstract

It is known that deformation in disordered materials such as metallic glasses and supercooled liquids occurs via the cooperative rearrangement of atoms or constituent particles at dynamical heterogeneities, commonly regarded as point-like defects. We show via molecular-dynamics simulations that there is no apparent relationship between atomic rearrangements and the local atomic environment as measured by the atomic-level stresses, kinetic and potential energies, and the per-atom Voronoi volume. In addition, there is only a weak correlation between atomic rearrangements and the largest and smallest eigenvalues of the dynamical matrix. Our results confirm the transient nature of dynamical heterogeneities and suggest that the notion of defects may be less relevant than that of a propensity for rearrangement.

## Introduction

Plastic deformation and structural relaxation in disordered materials such as metallic glasses, supercooled liquids, and granular matter occurs via the cooperative rearrangement of atoms or constituent particles at dynamical heterogeneities^1–13^. These clusters of atoms or particles are sometimes termed shear transformation zones (STZs) in the literature^14,15^, and have initially been associated with excess free volume^16–19^. Identification of these dynamical heterogeneities or STZs by features of the local atomic or particle environment, in the hopes of predicting when and where these stress- and thermally-activated rearrangement of particles will occur, has long been a fundamental challenge.

Manning and Liu^20^ extensively studied the low-frequency vibrational modes of a metallic glass and identified a population of structural soft spots where particle rearrangements are initiated. More recently, Refs.^21,22^ applied machine learning methods and found that a large and convoluted set of structure functions may be able to predict particle rearrangement. In both cases, however, the accuracy is far from perfect. In addition, the physical meaning of the structure functions used in^21,22^ is obscured by their complexity. A natural question arises: can simple measures of the local atomic environment help us predict where structural relaxation and particle rearrangements may take place in a disordered material?

In the present study, we focus on the atomic-level stresses, energies, Voronoi volume, and the local potential energy landscape, and investigate if there are relationships between these direct measures of the local atomic environment and the occurrence of atomic rearrangement. Our results indicate the apparent absence of correlation between the local atomic environment as measured by the atomic-level stresses, energies, and volumes, and the occurrence of local atomic rearrangements. In addition we find a weak correlation between the largest and smallest eigenvalues of the dynamical matrix, measures of the local potential energy landscape, and the occurrence of atomic rearrangements. Our results suggest that one must proceed with extreme caution when attempting to relate the notion of defects to dynamics; we will discuss the origin of such apparent lack of correlations and the implications of our findings towards the end of the paper.

## Results

In the following we only show results for $T = 300$ K, but similar results are seen at the higher temperatures of 800 and 1500 K.

**Figure 1:**
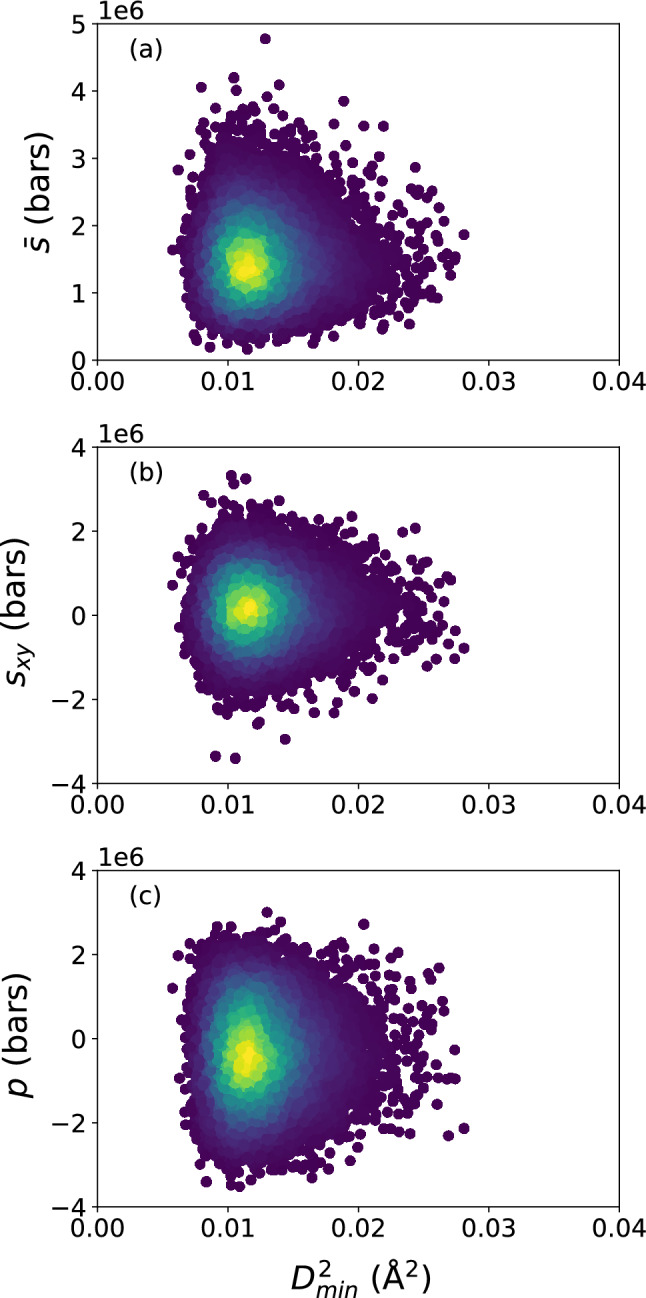
Scatter plots of (**a**) deviatoric stress invariant ${\bar{s}}$, (**b**) shear stress $s_{xy}$, and (**c**) atomic-level pressure *p*, versus $D_{\text {min}}^2$, for the $N = 16{,}000$ atoms, at the shear strain of 0.099, at a strain rate of ${\dot{\gamma }}_{xy} = 10^6$ s$^{-1}$. The colors indicate the density of points computed using a kernel-density estimate with Gaussian kernels, with dark purple representing the lowest density and yellow representing the highest density.

**Figure 2:**
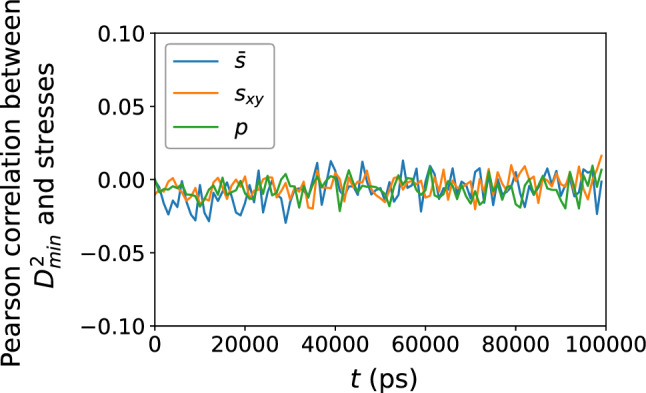
Pearson correlation between the local atomic stress measures ${\bar{s}}$, $s_{sy}$, and *p* versus $D_{\text {min}}^2$, confirming no apparent relationship between structural relaxation or deformation and the local atomic stress environment.

**Figure 3:**
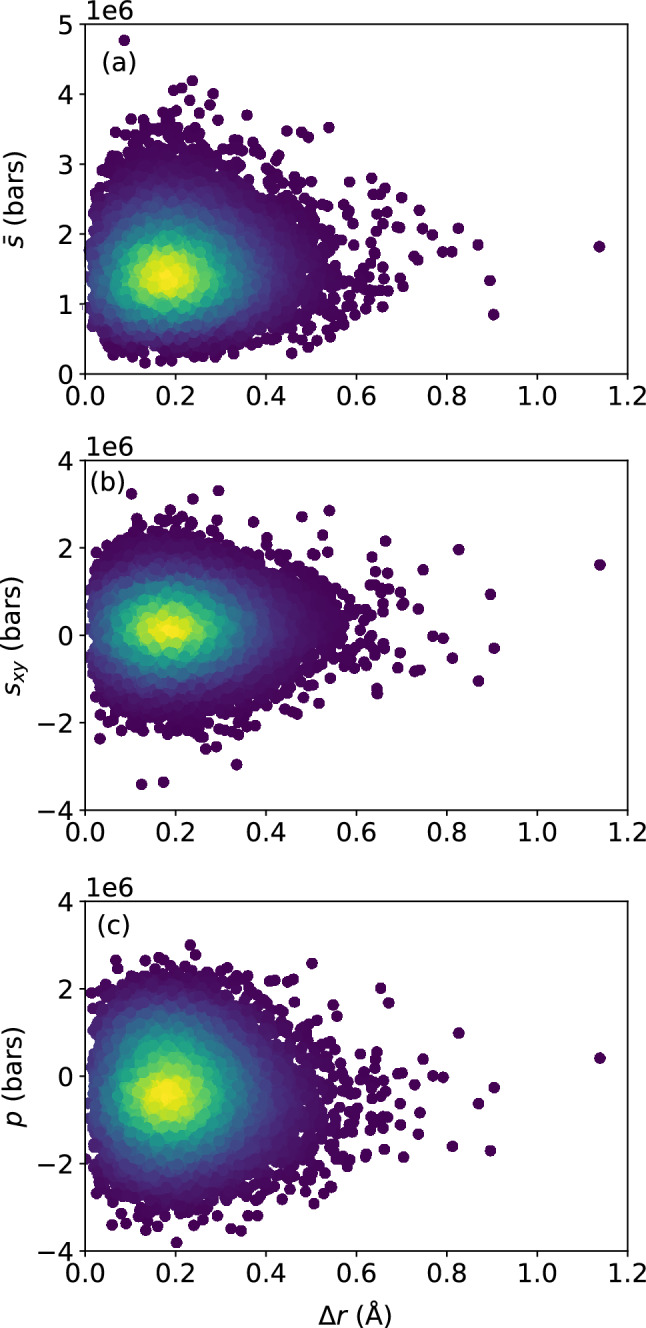
Scatter plots of (**a**) deviatoric stress invariant ${\bar{s}}$, (**b**) shear stress $s_{xy}$, and (**c**) atomic-level pressure *p*, versus $\Delta r$, for the $N = 16{,}000$ atoms, at the shear strain of 0.099, at a strain rate of ${\dot{\gamma }}_{xy} = 10^6$ s$^{-1}$. The colors indicate the density of points computed using a kernel-density estimate with Gaussian kernels, with dark purple representing the lowest density and yellow representing the highest density.

**Figure 4:**
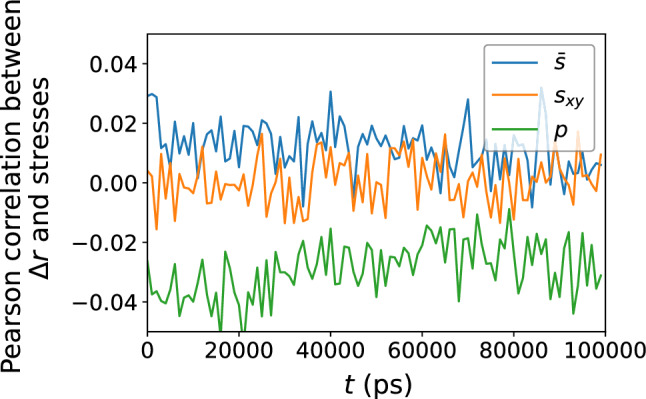
Pearson correlation between the local atomic stress measures ${\bar{s}}$, $s_{xy}$, and *p* versus $\Delta r$, confirming no apparent relationship between atomic displacement and the local atomic stress environment.

**Figure 5:**
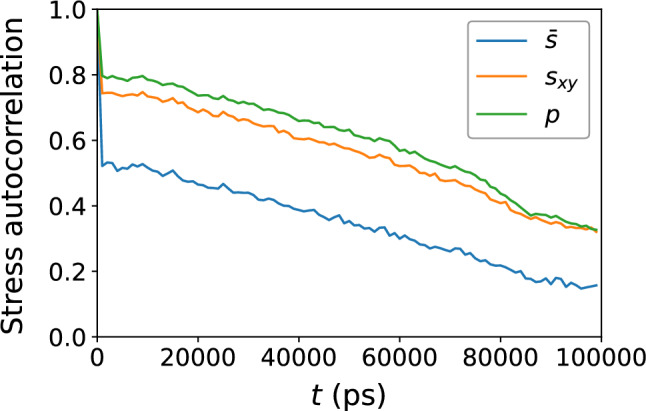
Autocorrelation the local atomic-level stresses ${\bar{s}}$, $s_{xy}$, and *p*. The decay shows that memory of the atomic-level stresses is gradually lost with time.

### Atomic-level stresses

Figure 1 shows the atomic-level stress measures ${\bar{s}}$, $s_{xy}$, and *p* versus the quantity $D_{\text {min}}^2$, which measures the nonaffine atomic displacement and describes the occurrence of plastic events, for the $N = 16{,}000$ atoms. (The reader may refer to the “Methods” section for the definitions of the stress measures ${\bar{s}}$, $s_{xy}$, and *p*.) Here we only show the snapshot at $t = 99{,}000$ ps, or a shear strain of $\gamma _{xy} = 0.099$, after the imposition of simple shear at ${\dot{\gamma }}_{xy} = 10^6$ s$^{-1}$, but the results at other time snapshots are similar. The scatter of the data points indicates no apparent relationship between the local atomic stresses and the occurrence of rearrangements; this is confirmed by Fig. 2, which shows a Pearson correlation coefficient between $-0.04$ and 0.04 for each of these local atomic stress measures and quantity $D_{\text {min}}^2$ over the duration of the simulation.

We can perform the same analysis with the atomic displacement $\Delta r$ over time increment $\Delta t$ instead of $D_{\text {min}}^2$; the result is shown in Figs. 3 and 4 . Once again, the local atomic stress measures bear little correlation with the atomic displacement over time $\Delta t$, and do not serve as a good predictor of the latter.

Figure 5 shows the decay of the stress autocorrelations for ${\bar{s}}$, $s_{xy}$, and *p*; all three show a gradual decay with the time range sampled. This shows that memory of the original atomic stress environment is gradually lost.

**Figure 6:**
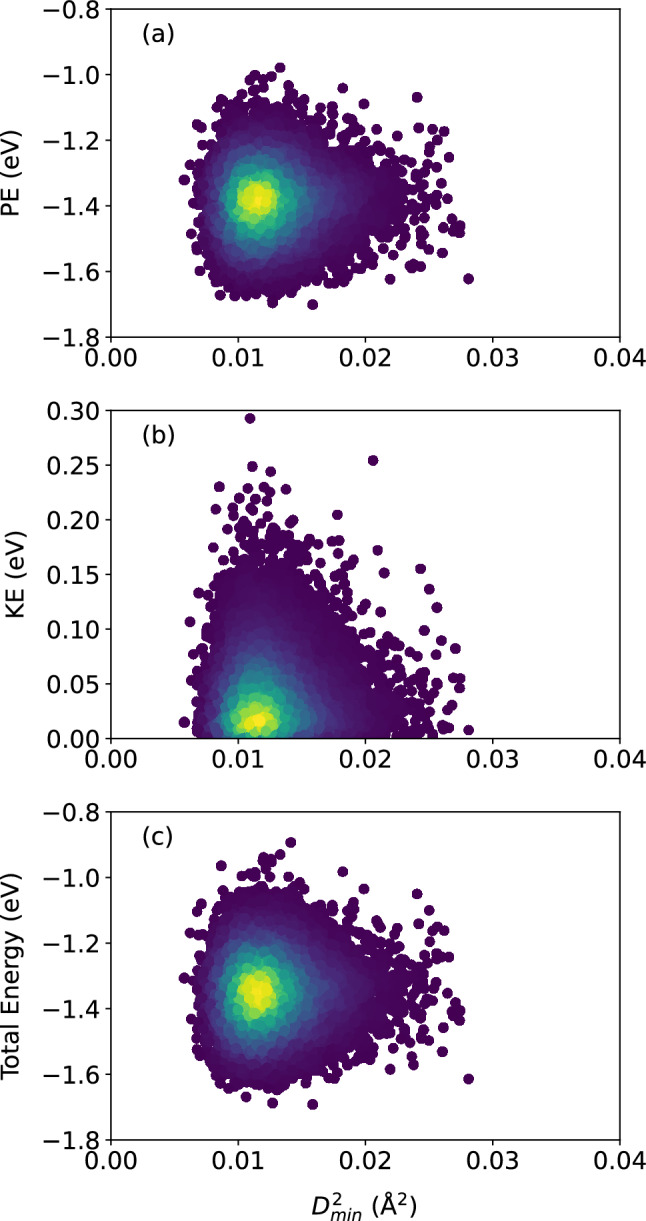
Scatter plots of (**a**) atomic potential energy, (**b**) atomic kinetic energy, and (**c**) their sum, versus $D_{\text {min}}^2$, for the $N = 16{,}000$ atoms, at the shear strain of 0.099, at a strain rate of ${\dot{\gamma }}_{xy} = 10^6$ s$^{-1}$. The colors indicate the density of points computed using a kernel-density estimate with Gaussian kernels, with dark purple representing the lowest density and yellow representing the highest density.

**Figure 7:**
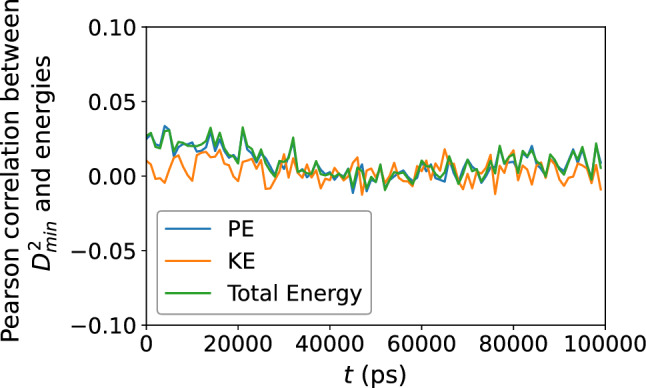
Pearson correlation between the atomic potential energy, kinetic energy, and their sum, versus $D_{\text {min}}^2$, confirming no apparent relationship between structural relaxation or deformation and the local atomic energies.

**Figure 8:**
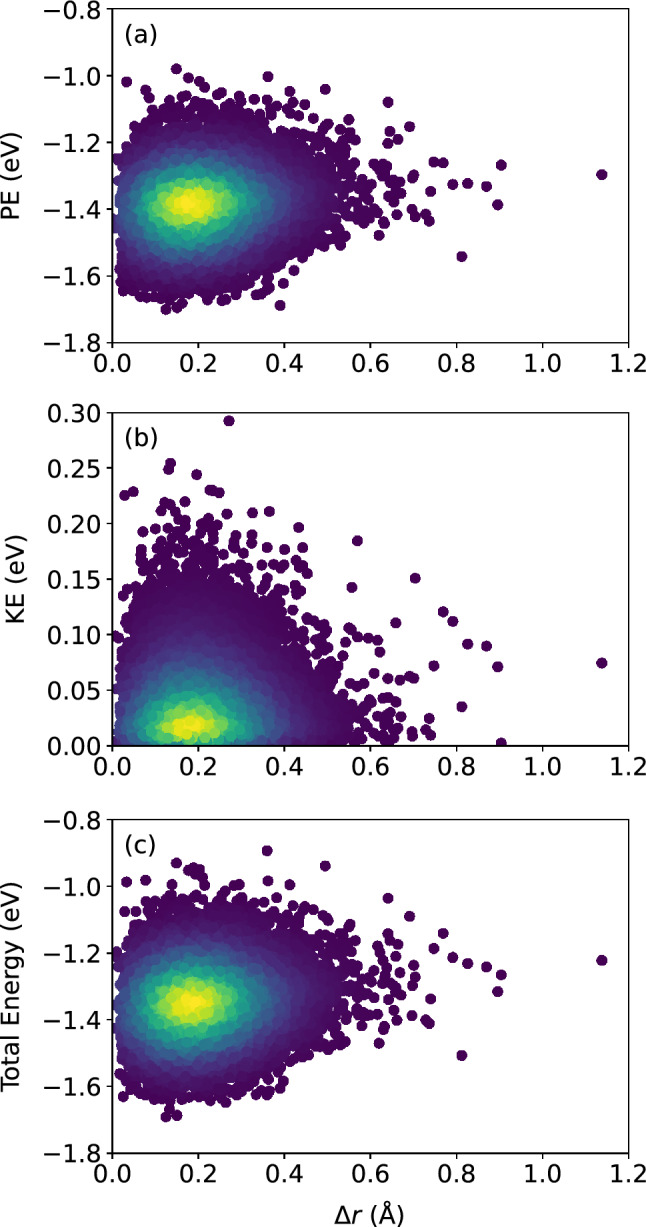
Scatter plots of (**a**) atomic potential energy, (**b**) atomic kinetic energy, and (**c**) their sum, versus $\Delta r$, for the $N = 16{,}000$ atoms, at the shear strain of 0.099, at a strain rate of ${\dot{\gamma }}_{xy} = 10^6$ s$^{-1}$. The colors indicate the density of points computed using a kernel-density estimate with Gaussian kernels, with dark purple representing the lowest density and yellow representing the highest density.

**Figure 9:**
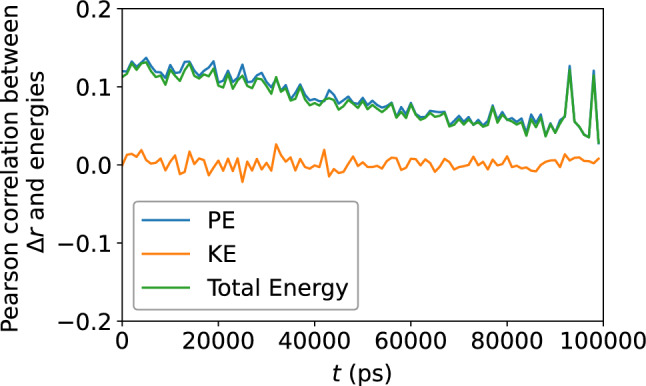
Pearson correlation between the atomic potential energy, kinetic energy, and their sum, versus $\Delta r$, confirming no apparent relationship between atomic displacement and the local atomic energies.

**Figure 10:**
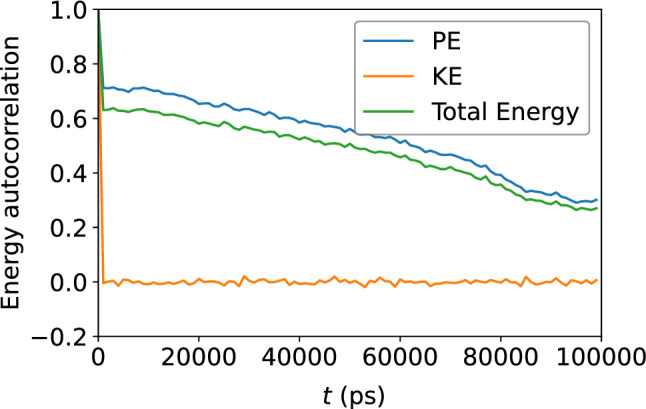
Autocorrelation the atomic potential energy, kinetic energy, and their sum. The decay shows that memory of the initial energies is gradually lost with time.

### Atomic-level energies

Turn now to the atomic energies. Figure  6 shows the atomic potential energy, kinetic energy, and their sum, versus $D_{\text {min}}^2$. Once again, the scatter of the data suggests that there is no apparent relationship between the atomic-level energies and the nonaffine displacement of each atom in the supercooled metallic liquid. This is corroborated by Fig. 7 which shows only a very weak correlation between each of the three energies and $D_{\text {min}}^2$. We repeat the same analysis in Figs. 8 and 9 and find no apparent correlation between atomic displacement and atomic-level energies. In addition, Fig. 10 shows that while the autocorrelations in the potential energy and total energy decay gradually, once again indicative of a slow loss of memory of the atomic environment, the autocorrelation of the kinetic energy decays very rapidly to zero.

**Figure 11:**
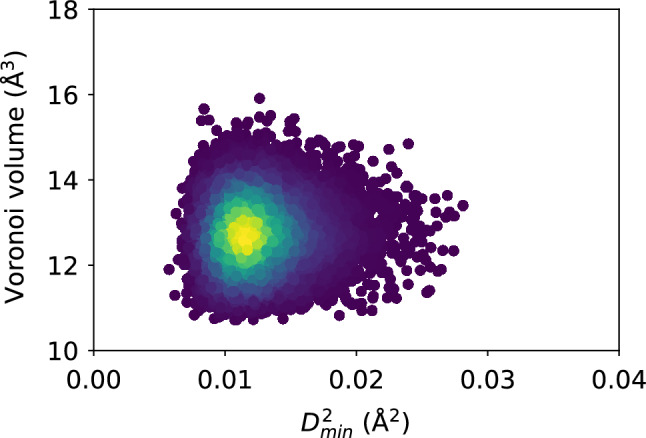
Scatter plots of Voronoi tessellation volume versus $D_{\text {min}}^2$, for the $N = 16{,}000$ atoms, at the shear strain of 0.099, at a strain rate of ${\dot{\gamma }}_{xy} = 10^6$ s$^{-1}$. The colors indicate the density of points computed using a kernel-density estimate with Gaussian kernels, with dark purple representing the lowest density and yellow representing the highest density.

**Figure 12:**
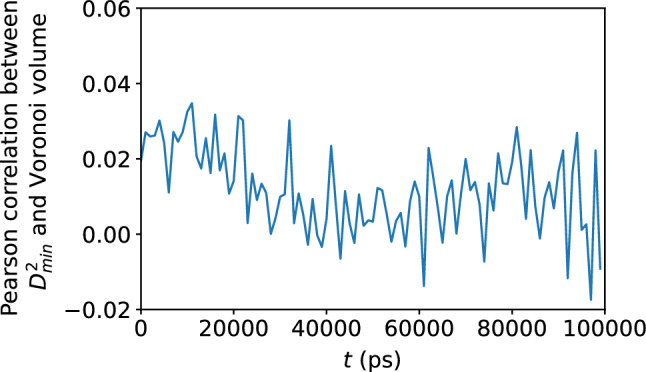
Pearson correlation between the Voronoi tessellation volume and $D_{\text {min}}^2$, confirming no apparent relationship between structural relaxation or deformation and the local atomic volume.

**Figure 13:**
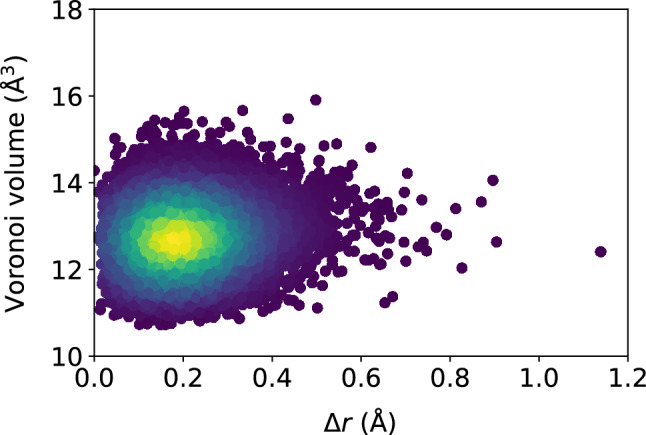
Scatter plots of the Voronoi tessellation volume versus $\Delta r$, for the $N = 16{,}000$ atoms, at the shear strain of 0.099, at a strain rate of ${\dot{\gamma }}_{xy} = 10^6$ s$^{-1}$. The colors indicate the density of points computed using a kernel-density estimate with Gaussian kernels, with dark purple representing the lowest density and yellow representing the highest density.

**Figure 14:**
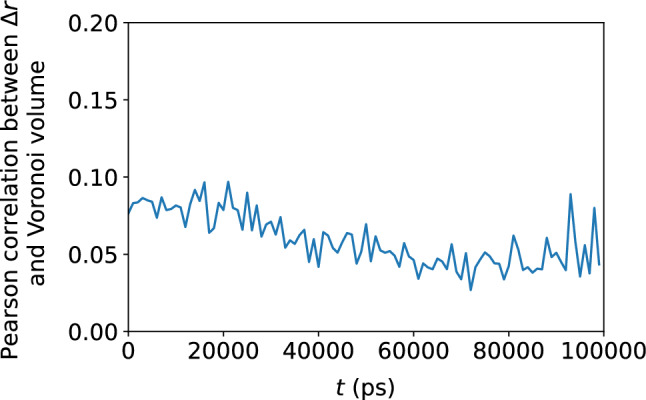
Pearson correlation between the Voronoi tessellation volume and $\Delta r$, confirming no apparent relationship between atomic displacement and the local atomic volume.

**Figure 15:**
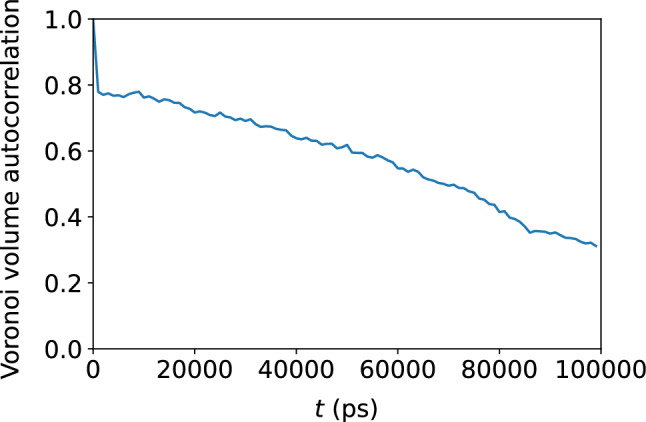
Decay of the autocorrelation the Voronoi tessellation volume with time, indicating gradual loss of memory of the original local atomic environment.

### Atomic-level volume

We show in Figs. 11 and 12 the very weak correlation between $D_{\text {min}}^2$ and the Voronoi tessellation volume, meaning that the free volume available to an atom is not a good predictor of nonaffine atomic rearragement. The analogous weak relationship between the atomic displacement $\Delta r$ and the atomic volume is shown in Figs. 13 and 14. The gradual loss of memory of the atomic volume is shown in Fig. 15.

### Dynamical matrix

**Figure 16:**
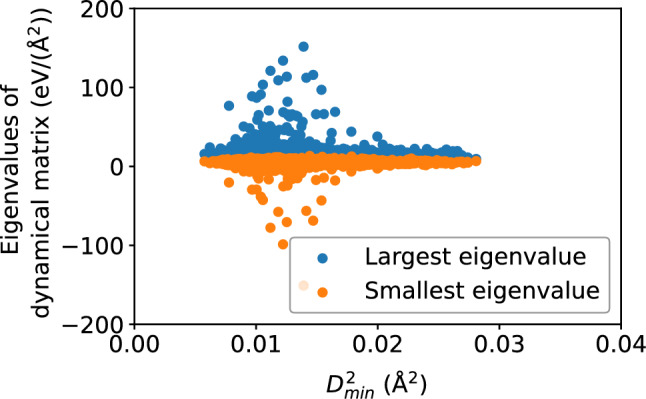
Scatter plots of largest and smallest eigenvalues of the dynamical matrix versus $D_{\text {min}}^2$, for the $N = 16{,}000$ atoms, at the shear strain of 0.099, at a strain rate of ${\dot{\gamma }}_{xy} = 10^6$ s$^{-1}$.

**Figure 17:**
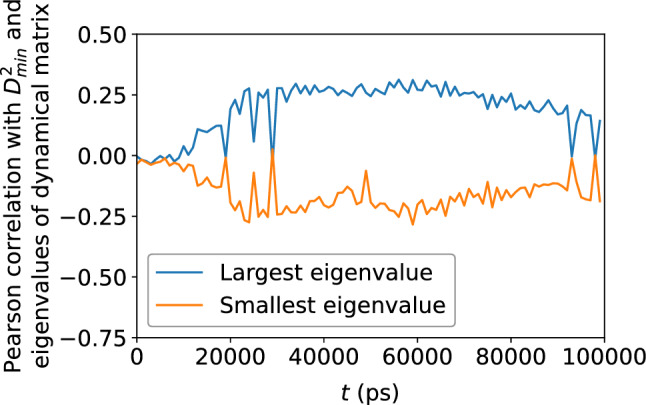
Pearson correlation between the largest and smallest eigenvalues of the dynamical matrix and $D_{\text {min}}^2$, showing a weak correlation between structural relaxation or deformation and the local potential energy landscape.

**Figure 18:**
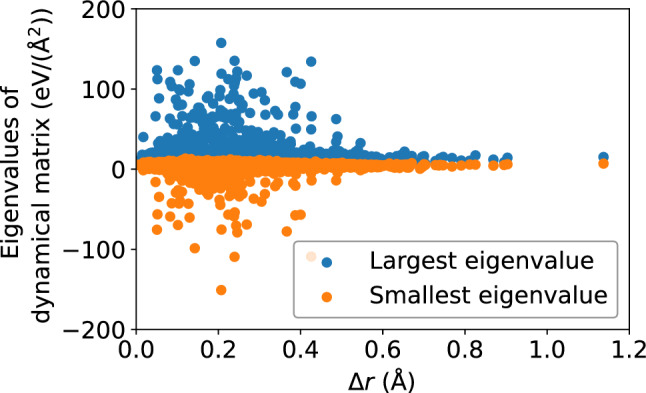
Scatter plots of the largest and smallest eigenvalues of the dynamical matrix versus $\Delta r$, for the $N = 16{,}000$ atoms, at the shear strain of 0.099, at a strain rate of ${\dot{\gamma }}_{xy} = 10^6$ s$^{-1}$.

**Figure 19:**
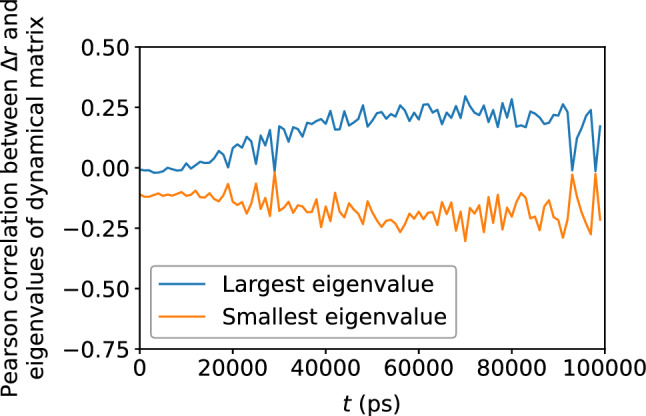
Pearson correlation between the largest and smallest eigenvalues of the dynamical matrix and $\Delta r$, depicting a weak correlation between atomic displacement and the local potential energy landscape.

**Figure 20:**
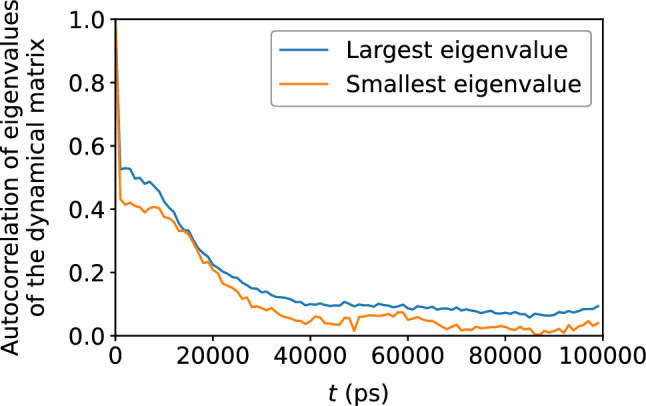
Decay of the autocorrelation the Voronoi tessellation volume with time, indicating gradual loss of memory of the original local atomic environment.

The dynamical matrix, computed in accordance with Eqs. (5) and (6), measure the local potential energy landscape; in particular, its eigenvalues measure the curvature of the potential energy landscape. Figure 16 shows a scatter plot of the largest and smallest eigenvalues of the dynamical matrix versus $D_{\text {min}}^2$; Fig. 17 shows that the correlation between the largest eigenvalue and $D_{\text {min}}^2$, as well as the anti-correlation between the smallest eigenvalue and $D_{\text {min}}^2$, both increase with accumulated shear strain, but never exceeds 0.4, implying a somewhat weak relationship between the potential energy landscape and nonaffine displacement. We see roughly the same relationship in Figs. 18 and 19 between the potential energy landscape and the atomic displacement $\Delta r$. Finally, Fig. 20 shows that the autocorrelation of the extremal eigenvalues of the dynamical matrix decays rapidly with time—more so when compared to the atomic-level stresses and energies—and implies the transient nature of the local potential energy landscape as atoms are displaced.

## Discussion

We have seen in this paper that simple measures of the local atomic environment, such as the atomic-level stresses, kinetic and potential energies, the atomic volume, and the potential energy landscape, do not serve as good predictors of the subsequent nonaffine displacement of an atom in a disordered material. As such, the idea that one can regard dynamical heterogeneities and STZs as defects is in serious question. In crystalline solids the defects, such as vacancies, maintain their identities after movement, because they are topologically protected by the lattice periodicity. In the absence of the lattice, however, the local structures in glasses keep changing during mechanical deformation. Consequently, the local structure initially identified as a defect changes during the deformation event. In fact, by the time the system reaches the saddle point in the potential energy landscape, the previous thermal history is nearly wiped out^23^, because it locally melts configurationally for a very short time^24^. Therefore, it is more likely that the mechanical properties of metallic glasses are controlled by the average structure, characterized by the effective temperature of STZ^14,15^ or the medium-range order^25^, than local defects.

This does not mean, however, that the notion of dynamical heterogeneities and STZs are useless. Rather, our results illustrate the transient nature of these flow heterogeneities. Namely, during the deformation event an STZ fluctuates into existence, may or may not perform a cooperative rearrangement, and then disappears^26^. As such, plastic deformation and structural relaxation at these heterogeneities should be regarded as dynamic stochastic events whose propensity cannot be predicted solely by the local atomic environments, but is governed by the mesoscale statistical and thermodynamic properties of the material^15^. Our observations reaffirm the results recently described in^27^, which demonstrate that nearly constant loss in sinusoidally-driven metallic glasses is also stochastic in nature and does not occur at local structural defects. In other words, one must be cautious in relating structural relaxation to structural defects in disordered materials. Our findings complement prior studies on dynamical heterogeneities^1–13^, which showed the complex, highly cooperative nature of local atomic dynamics during deformation events. Here, we emphasize the dynamical nature of deformation even further, by pointing out that much of the features of the initial structure, such as the atomic energies (kinetic, potential, and total) and stresses (von Mises, shear stress), are poorly correlated with the features of atomic displacements, $\Delta r$ and $D_{\text {min}}^2$, because the initial structure is strongly modified during the deformation event. As such, our findings present a strong case for the apparent absence of a direct relationship between deformation and defects in glassy materials.

Our view is indirectly but strongly supported by experimental data. For instance, the mechanical strength of metallic glasses is universally related to the elastic constants and not to the details of sample preparation^28,29^. If defects were to blame for mechanical deformation, strength would be very sensitive to the way samples are prepared, and its universal dependence on elastic constants would not be expected.

In summary, we show, through molecular dynamics simulation, that local deformation events in metallic glasses occur nearly randomly, with little or no correlation with the local atomic environment. The results suggest that mechanical deformation in metallic glass is highly stochastic, and the notion of defects may not be so relevant, unlike in crystalline solids.

## Methods

**Figure 21:**
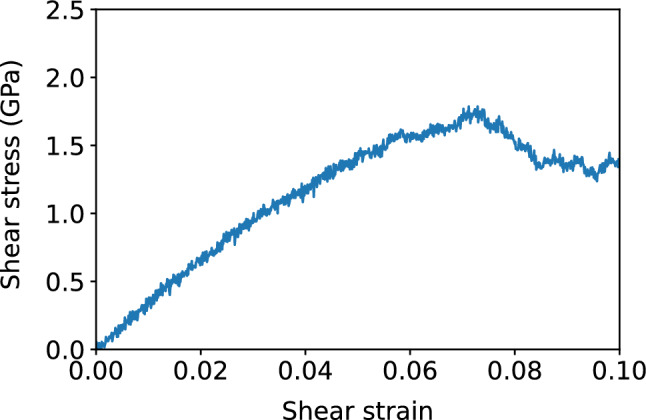
Stress-strain curve of supercooled Fe at a temperature of $T = 300$ K undergoing simple shear, at a strain rate of ${\dot{\gamma }}_{xy} = 10^6$ s$^{-1}$.

We performed molecular dynamics simulations using the Large Scale Massively Parallel Simulator (LAMMPS)^30^. The supercooled Fe sample was prepared with a melt-quench method, starting with $N = 16{,}000$ Fe atoms, interacting with the modified Johnson potential and in a bcc lattice with a cubic side length of 58.86 Å, melted at 5000 K, with periodic boundary conditions. The sample was then supercooled at a rate of $10^3$ K/ps in an NVT ensemble and equilibrated over 1 ns, in intervals of 100 K, down to a temperature of 1500 K. Then it was supercooled over 0.1 ps to a temperature of 800 K or 300 K, and equilibrated over 1 ns. This was to prevent crystallization which could occur at temperatures between 1000 and 1300 K. At temperature $T = 1500$ K, 800 K, and 300 K, we performed simple shear on the supercooled Fe sample, at a strain rate of ${\dot{\gamma }}_{xy} = 10^6$ s$^{-1}$, with a time step size of 0.001 ps, up to a shear strain of 0.1, or total time of $10^5$ s. which is in the post-yield regime. This is illustrated by the stress-strain curve in Fig. 21.

We compared atomic positions at a time interval of $\Delta t = 1$ ps. We computed the atomic displacements for each atom *i*, $\Delta r_i = |{\textbf{r}}_i (t + \Delta t ) - {\textbf{r}}_i (t) |$, as well as the quantity^14^

1 $$\begin{aligned} D_{\text {min},i}^2= & {} \min _{\Lambda } \dfrac{1}{z} \sum _j \left[ ({\textbf{r}}_i (t + \Delta t) - {\textbf{r}}_j (t + \Delta t) ) \right. \nonumber \\ {}{} & {} \left. - \Lambda ( {\textbf{r}}_i (t) - {\textbf{r}}_j (t) ) \right] ^2 \end{aligned}$$

which characterizes the nonaffine atomic displacement of each atom *i* over the time interval $\Delta t$. The sum in Eq. (1) is performed over all *z* neighbors *j* within a cutoff distance of 5.44 Å (i.e., the cutoff of the modified Johnson potential plus 2 Å) from the atom *i*, but the choice of the cutoff distance is inessential. The tensor $\Lambda$ which minimizes $D_{\text {min}}^2$ can be solved analytically^14^ but it is easier to compute $D_{\text {min}}^2$ directly by least-squares minimization in practice. The results are not sensitive to our choice of $\Delta t$. We then relate the $\Delta r$ and $D_{\text {min}}^2$ computed for each atom at time *t* with the atomic-level stresses, energies, and volume. Specifically, given the interatomic potential $\phi (r)$, the atomic-level stress tensor $\sigma _{\alpha \beta }^i$ of atom *i* is computed by summing over all of its neighbors *j*, as follows^31^:

2 $$\begin{aligned} \sigma _{\alpha \beta }^i = \dfrac{1}{2 v_i} \sum _j \dfrac{1}{r_{ij}} \dfrac{d \phi }{d r_{ij}} r_{ij}^{\alpha } r_{ij}^{\beta }, \end{aligned}$$

where ${\textbf{r}}_{ij} \equiv {\textbf{r}}_i - {\textbf{r}}_j$, and $v_i$ is the volume of atom *i*. We restrict ourselves to the deviatoric stress invariant

3 $$\begin{aligned} {\bar{s}} = \sqrt{\dfrac{1}{2} s_{\alpha \beta } s_{\alpha \beta }}; \quad s_{\alpha \beta } \equiv \sigma _{\alpha \beta } - \dfrac{1}{3} \delta _{\alpha \beta } \sigma _{\gamma \gamma }, \end{aligned}$$

the shear stress component $s_{xy}$ in the direction of the shear, and the pressure

4 $$\begin{aligned} p = - \dfrac{1}{3} \sigma _{\alpha \alpha }. \end{aligned}$$

We include the kinetic and potential energies of each atom as well as their sum. The atomic volume $v_i$ is computed by Voronoi tessellation using the Voro++ package^32^. In addition, we perform a normal mode analysis and compute, for each atom *i*, its $3 \times 3$ dynamical matrix^33^

5 $$\begin{aligned} H_{ii}= & {} \dfrac{\partial ^2 U}{\partial {\textbf{r}}_i \partial {\textbf{r}}_i} = \sum _j M_{ij}; \end{aligned}$$

6 $$\begin{aligned} M_{ij}= & {} u'' (r_{ij}) {\textbf{n}}_{ij} {\textbf{n}}_{ij}^T + \dfrac{u' (r_{ij})}{r_{ij}} (I - {\textbf{n}}_{ij} {\textbf{n}}_{ij}^T ), \end{aligned}$$

where the sum is over all atoms *j* within the cutoff distance of 5.44 Å, *U* is the total potential energy, $u(r_{ij})$ is the pair interaction potential, and ${\textbf{n}}_{ij}$ is the unit vector in the direction from atom *i* to atom *j*. The eigenvalues of the dynamical matrix measure the curvature of the local potential energy landscape and, in turn, provide another descriptor of the local atomic environment.

## Data Availability

The simulation data are available from the corresponding author upon reasonable request.
